# Supplementary material for: The impact and cost of a new rapid diagnostic test for school-based prevalence mapping and monitoring and evaluation surveys of schistosomiasis: A modelling study
Source: PLoS Negl Trop Dis. 2025 May 12;19(5):e0013071. doi: 10.1371/journal.pntd.0013071 (PMC12097705; doi:10.1371/journal.pntd.0013071)

# **S1 Text**

## **Costing**

Description of the costing model:

1. *Kato-Katz and urine filtration*

This testing strategy involves evaluating selected SACs for *S. mansoni* or *S. japonicum* infection using one stool sample collected at the school, which is then evaluated using microscopes offsite. Two slides are prepared for each stool sample by a field assistant, totaling two slides for each SAC. The slides are evaluated by Kato-Katz thick-smear examination and read by two laboratory technicians. Results are reported to schools on a separate trip. This model assumes that stools can be collected from an average of two schools per day, the field assistant can prepare 250 slides per day, and laboratory technicians can read 50 slides each per day. Total days in the field are calculated by taking the number of school visits (which included result delivery visits) and dividing it by the number of schools that could be visited in a day. Result delivery visits were calculated at half the time of normal screening visits. Individuals are considered infected if either of the two slides is positive for *S. mansoni* or *S. japonicum* eggs. In addition, urine filtration was used to evaluate the urine of selected SACs for *S. haematobium* infection using one urine sample collected at the school, which is first evaluated with haemastix and then further evaluated by a laboratory technician via microscopy offsite.

1. *CAA RDT*

Selected SAC are evaluated for *S. mansoni*, *S.japonicum* and *S.haematobium* infection using a single finger-prick blood sample collected at the school by a laboratory technician and read by a field assistant. The POC-CAA testing is performed at a central location in the village according to the test developer, DCN ([www.dcndx.com](http://www.dcndx.com)). The test is read and results recorded by the field assistant or laboratory technician after a 20-minute development time. This model assumes that two villages can be evaluated per day. Individuals are considered infected if the test is positive according to manufacturer’s instructions.

All models include the labor contribution of a field coordinator who supervises the work and coordinates the field team, and a driver, who drives the team between the schools and the offsite laboratory.

**Table A**. Cost categories and resource use and prices

| Cost category | Description |
| --- | --- |
| Personnel costs | A daily rate was calculated for laboratory technicians, field assistants, drivers and programme coordinators using total compensation and assuming 20 working days per month(25). Total personnel costs were calculated by multiplying the per day rates by the number of field days (for field staff) and the number of person-days required to evaluate the number of slides (for laboratory staff). |
| Supplies | Supplies include field and laboratory consumable supplies required to conduct the respective tests. Costs for the consumables were obtained from the CAA SCH field evaluation, or publicly available sources and units required per test were sourced from expert opinion. Total supply costs were determined by multiplying out by the number of samples and schools included. The price of a CAA RDT was taken as $3 as per the TPP requirements. Shipping and distribution fee of 15% was used. |
| Capital and training | This included capital items such as laboratory equipment (e.g., microscopes), laboratory durable goods (e.g., storage containers), field-based durable goods (e.g., cool boxes, ice packs), and other capital goods such as laptops. A one-day staff training was also included. Fixed costs were annualized over their useful working life of the asset and a per day cost was determined and multiplied by the number of field days. |
| Transport | This included a vehicle rental rate per day, a fuel cost per day, and a fixed cost to drive the vehicle to the field from a central location (Nairobi). The per day costs were multiplied out by the number of field days (as well as the result delivery days in the case of Kato-Katz). These costs were based on project expenditure from the Schistosomiasis CAA RDT field study in Kenya. |
| Overhead | To account for the indirect costs associated with the administration and management of the survey, such as buildings, facilities, administrative support, etc., we applied a fixed overhead percentage (18%) as routinely applied to KEMRI projects. |

To account for the fact that transport and staffing costs could be more or less expensive in other settings/regions, these costs were varied. Geographic accessibility is characterized in terms of the number of schools that can be visited a day: an increase from two to three in small, densely populated enumeration units. Geographic accessibility is also characterized in terms of the transport costs—lower costs (-50%) per day for smaller geographic areas or more densely populated regions. For the minimum staffing costs, we reduced the daily rate by 50%. When staffing costs are halved, Kato-Katz costs less than the CAA RDT when traditional sampling is used and costs less than CAA RDT in SPPA Stage 1 sampling only when transport costs are also halved (Figure S1).

**Fig A**. Percentage cost difference between Kato-Katz and a CAA RDT testing strategy with varying staff costs, and transport costs

## **Minimum requirements**

**Table** **B**. Minimum sensitivity and specificity to achieve greater than or equal to 80% correct sub-district treatment, by district archetype and sampling strategy.

| Prevalence | Distribution | SPPA Sampling | | Traditional Sampling | |
| --- | --- | --- | --- | --- | --- |
|  |  | Sensitivity | Specificity | Sensitivity | Specificity |
| 5% | **Homogeneous** | 60% | 96% | 60% | 95% |
| 8% |  | 60% | **98%** | 60% | **97%** |
| 12% |  | **60%** | 95% | **65%** | 95% |
| 20% |  | 60% | 95% | 60% | 95% |
| 5% | **Heterogeneous** | 60% | 96% | 60% | 97% |
| 8% |  | 60% | **99%** | ­— | — |
| 12% |  | **—** | — | — | — |
| 20% |  | — | — | — | — |
| All | **Homogeneous** | 60% | 97% | 60% | 97% |
|  | **Heterogeneous** | 60% | 98% | — | — |
| Total | | 60% | 97% | — | — |

**Bimodal Prevalence Sensitivity Analysis**

**Fig B**. Percent of sub-districts that were classified for overtreatment when average prevalence was <10% (A) or undertreatment when average prevalence was >10% (B), by sampling strategy, test sensitivity, and specificity, in three districts with bimodal prevalence distributions (8%—7 districts 5% prevalence, 3 districts 15% prevalence, 10%—5 districts 5% and 5 districts 15% prevalence, 12%—3 districts 5% prevalence, 7 districts 15% prevalence). The dashed line at 0% represents 100% correct sub-district classification.


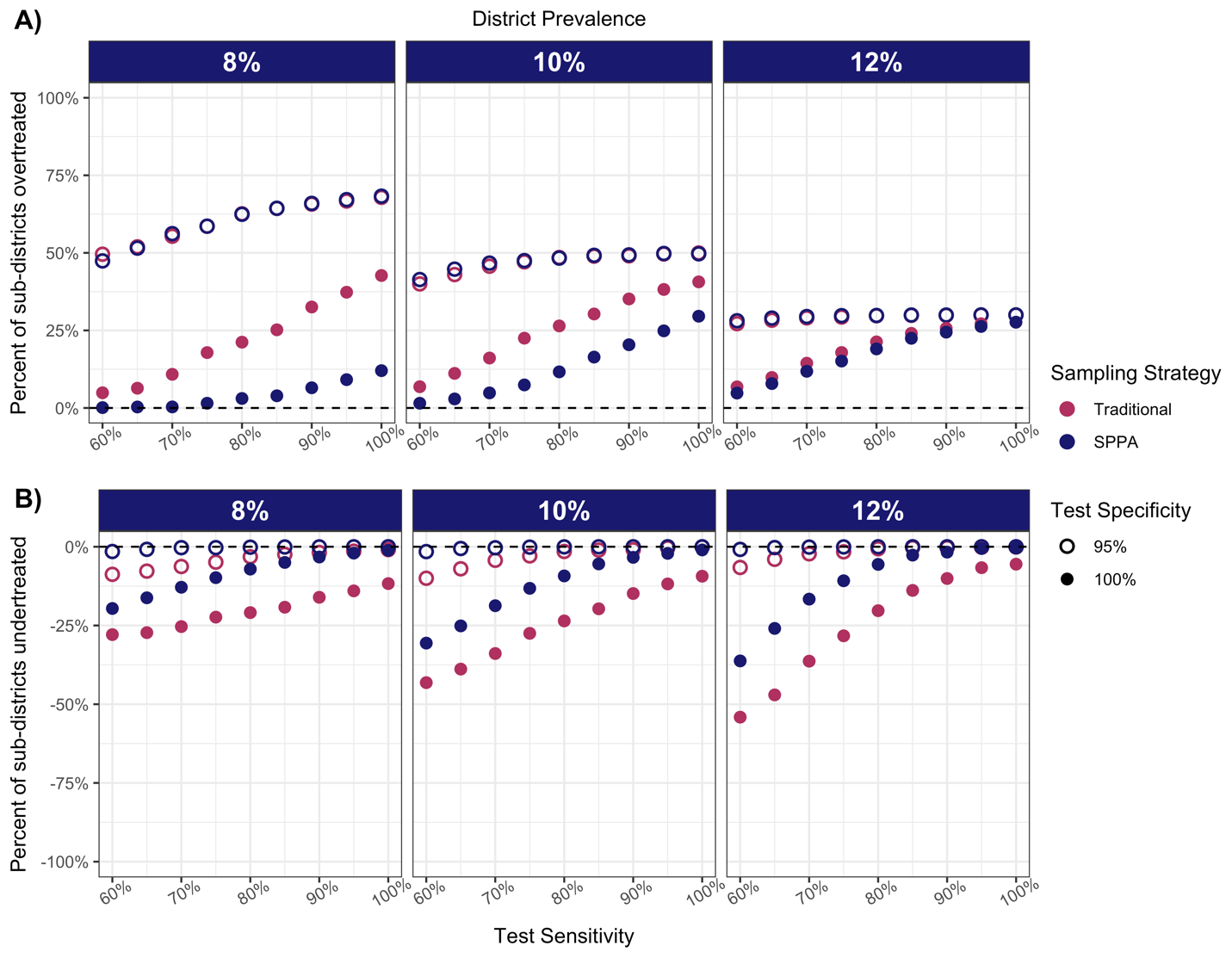

Supplement: S1 Text — Additional costing methodology and sensitivity analyses. Fig A. Percentage cost difference between Kato-Katz and a CAA RDT testing strategy with varying staff costs, and transport costs. Fig B. Percent of sub-districts that were classified for overtreatment when average prevalence was < 10% (A) or undertreatment when average prevalence was > 10% (B), by sampling strategy, test sensitivity, and specificity, in three districts with bimodal prevalence distributions. Table A. Cost categories and resource use and prices. Table B. Minimum sensitivity and specificity to achieve greater than or equal to 80% correct sub-district treatment, by district archetype and sampling strategy. (DOCX) [file pntd.0013071.s001.docx]
