# Supplementary material for: The impact and cost of a new rapid diagnostic test for school-based prevalence mapping and monitoring and evaluation surveys of schistosomiasis: A modelling study
Source: PLoS Negl Trop Dis. 2025 May 12;19(5):e0013071. doi: 10.1371/journal.pntd.0013071 (PMC12097705; doi:10.1371/journal.pntd.0013071)
Supplement: Attachment — Submitted filename: Reviewers comments PNTD-24-01659-1.docx [file pntd.0013071.s002.docx]

**Reviewer’s comments**

**Title** Evaluating the impact and cost of a new rapid diagnostic test for school-based prevalence mapping and monitoring and evaluation surveys of Schistosomiasis: a modeling study

**Manuscript Number**: PNTD-D-24-01659

The authors have submitted an interesting piece of work focusing on the impact and cost of a novel rapid diagnostic test for schistosomiasis against standard microscopy and thereby evaluating its use in a precision mapping and monitoring strategy.

**Comprehensive Summary**

The manuscript provides an extensive analysis of the impact and cost of a novel rapid diagnostic test for schistosomiasis against standard microscopy and thereby evaluating its use in a precision mapping strategy. The authors revealed that CAA RDT in comparison with the conventional diagnostic methods (Kato-Katz and urine filtration) has shown high specificity and cost-effectiveness thereby leading to similar treatment success at lower costs, under either the SPPA or traditional sampling methods. Based on their results they attempted to suggest the possible shift of traditional diagnostic approaches to better sensitive and specific methods used in prevalence mapping of schistosomiasis rendering a significant input for the aspired elimination strategy. On the whole, the manuscript is meticulously organized, and the figures and tables provide corroborative material.

**Specific and minor comments**

Perhaps the authors should consider the following minor and/or line by line comments:

- The manuscript has no such a significant editorial problems to be addressed.
- Misplacement of items like objective under methods part is not common practice unless the rule of target journal. Otherwise, objectives are indicated implicitly under the last paragraph of the introduction.
- Scientific names should strictly be italicized and with consistent spacing throughout the manuscript. see abstract (results) and pp.14 Ln # 311
- Revise some abnormally long sentences Ln # 46-49, 97-100, 331-334, 370-373.
- Mixed referencing style (author-year Vs numeric citation style). Address those inconsistencies of citations in the text. See pp. 15, 16, 17 Ln # 356-357,361,386-387. Should be as follows: Knowles et al. (23)…, Finally, Worrell et al. (3) as cited in Ln#151…
- References are relatively too few. Hence, pertinent and up-to-date references should be added to enhance the visibility of the study.
- Improve the quality and clarity of figure 3 with regard to labeling. Table and Figure legends should be self-standing. Table legends do not need full stop see Table 1.
- Please check and follow the reference pattern as per journal and also balance of references in text Vs reference site.

**Title:** It is acceptable in its existing form but better if redundancy of using ‘evaluation’ is omitted.

**Abstract:** well-presented

**Introduction**: Sounds fine. It is well-presented.

**Methods**

This part is well-organized and more elaborative component of the manuscript. However, study setting and study duration were not described included. The authors should portray specifically where and when the study was conducted. Actually, the specific sites of the study should be elaborated in each sampled schools. The authors have redundantly been using the terms ‘district/sub-district’ throughout the manuscript. What are they referring to with these terms? Perhaps for such ambiguous terms and others, authors may be expected to add operational definition of terms.

It’s not clear why objective of the study was reflected under this part (Ln#121-128). Unless this is the format of the target journal, implicit description of the objective under the last paragraph of the introduction part seems adequate.

**Results and Discussion**

Generally, the report efficiently correlates results with current few literatures, so boosting the study's scientific merit.

The results are well presented and effectively highlighting the comparison of an alternative approach with the conventional diagnostic methods. The issue of cost effectiveness has been the major pillar in disease control including schistosomiasis and the present findings have underscored this scenario. The results were so comprehensive that the outcome might be translated into practice for schistosomiasis elimination policy in sub-districts and specific hot spot transmission sites across the world. On the other hand, overemphasizing of data (numerical data presentation both in text and table/fig.) has been observed in this part. So, authors need to avoid duplicate data presentation. Please check and at least minimize all duplicate presentations both in text and table/figure.

The discussion part was well organized but less emphasis was given on associating similar/related previous reports. The authors were obsessed in interpreting the outcome of their data hich is not discussion. No single citation was there in the first three paragraphs. See paragraph 1, 2 and 3 Ln# 299-336.

- For instance, the first paragraph is purely method part. So, needless to keep it.
- The second paragraph is partly a repetition of results with minimum interpretations.
- The remaining paragraphs are well written.

**Conclusions**

The conclusion looks interesting. These findings suggest promising innovative diagnostic approach after effective field performance evaluation is practiced. However, I am wondering that why the authors reserve performance evaluation of CAA RDT in the field for recommendation. It would have been an excellent work and complete picture if field performance evaluation of the diagnostic tool was conducted and complemented to this piece of work.

**References**

All references and citations should be checked for conformity with journal guidelines for authors.

**My opinion**

- Overall, the manuscript is well-structured, with a clear methodology and language command as well as compelling results, making a valuable contribution in mapping of schistosomiasis for possible elimination of the disease. I recommend acceptance with minor revision.

**Name of Reviewer**: Bayissa Chala (PhD, Associate Prof. of Parasitology & Tropical Medicine)

**Date**: December 14, 2024

Thank you!
