# Supplementary material for: The impact and cost of a new rapid diagnostic test for school-based prevalence mapping and monitoring and evaluation surveys of schistosomiasis: A modelling study
Source: PLoS Negl Trop Dis. 2025 May 12;19(5):e0013071. doi: 10.1371/journal.pntd.0013071 (PMC12097705; doi:10.1371/journal.pntd.0013071)
Supplement: Attachment — Submitted filename: Reviewer_Responses_PLOSNTD_2025Jan27_JMC_SG_KHG.docx [file pntd.0013071.s004.docx]

06 February 2025

Dear Editor and Reviewers,

On behalf of all authors, we would like to thank the reviewers and the editorial team for the opportunity to revise our manuscript and resubmit to *PLOS Neglected Tropical Diseases.* Below you will find our responses bulleted in **blue** addressing all reviewer comments by section of the manuscript. Please note that line numbers correspond to the clean version of the revised manuscript, not the marked copy.

**Methods**

*Reviewer 1****:***

Why repeat the Objective in the methods?

- As multiple reviewers have pointed out the repetition of the study objective in the methods, we have removed this restatement and have clarified the objective at the end of the introduction section (lines 118–124).

Line 140-142. No detailed CAA RDT procedure has been provided.
See Ref: <https://doi.org/10.3389/fpara.2024.1460331>

- We thank the reviewer for sharing this resource. As the CAA RDT is not yet commercially available for use, we made no assumptions regarding the testing technology other than the test sample (finger-prick capillary whole blood sample) and the time to test result (20 minutes). The use of the CAA RDT is hypothetical in this modelling analysis and no testing was conducted. However, the CAA technology would not impact our main outcomes. We assumed a per test kit cost of US$3 to align with the WHO target product profile (*Diagnostic target product profiles for monitoring, evaluation and surveillance of schistosomiasis control programmes*), while varying test sensitivity and specificity.

No information on the geographic distribution of targeted districts is provided. What’s the sizes of these districts? Can results from 5 schools (50 SAC per school) represent the prevalence of the district?

- The district archetypes in this modelling analysis are mathematically simulated as data frames in R and therefore do not represent actual districts with respect to geographical size or features. A district is synonymous with a county (or administration level two) and a sub-district is synonymous with a sub-county (administration level three). The SCH prevalence ranges of the district archetypes and the size of the districts (with respect to the number of sub-districts and schools per sub-district) were parameterized to publicly available data from Kenya but are intentionally meant to be generalizable archetypes applicable to other moderate- and high-burden settings. The district size in this analysis would correspond to a moderately sized district of one-million people, divided into 10 sub-districts of roughly 100,000 people each (based on 2019 census data). We assume 20% of the population (20,000/sub-district) is school-aged children, divided into schools of an average size of 370 (based on the Kenya Demographic and Health Survey of 2022 and the Basic Education Statistical Booklet of 2020), rounded to 50 schools per sub-district. We have added an additional statement with references to the methods section (lines 198–199).

We also thank the reviewer for bringing up such an important question. The sampling strategy of 5 schools and 50 SAC per school which we refer to as ‘traditional sampling’ is derived from previous WHO guidelines as referenced in the manuscript (line 175). As we show in **Figure 4**, this strategy is more likely to over-estimate prevalence rather than underestimate prevalence across the analyzed contexts. This is probably the result of the purposive sampling used in this strategy that is discussed in the next comment.

Lines 166-7, Why sites are selected purposively, not randomly, meaning sites with a higher potential for SCH (i.e. proximity to water bodies or known sites of transmission) are selected preferentially.

- As per the traditional sampling procedure outlined by the WHO, as referenced in the manuscript, schools are selected purposively. We have clarified this in the methods section (line 176). We presume schools are selected purposively through this methodology so as not to under-estimate prevalence in a district and therefore under-treat the district, which would be more harmful than over-treating a district.

Line 167-8, As a proxy for purposive sampling, we used a sampling weight proportional to school prevalence. Could you provide more details on this?

- Yes, we would be happy to clarify this point. The sampling weight used as a proxy for purposive sampling was calculated for each simulated school by dividing the prevalence of that school by the maximum school prevalence in the simulated district. This sampling weight then served as the probability for selection in the modelled sampling strategies. By this design, schools with higher prevalence have a higher probability of selection, simulating purposive sampling. We have added additional language to the methods to clarify this for the reader (lines 176–178).

Line 172, what’s the standard for a homogeneous/heterogeneous distribution in a district?

- There is not necessarily a standard for what constitutes homogeneous or heterogeneous distribution of prevalence. In a homogeneous district, site prevalences will be similar to one another, while in a heterogeneous district site prevalences could vary widely. We reference several prevalence studies conducted in Kenya that show how widely prevalence can range (line 205). These studies informed the simulation of district prevalence distributions outlined in **Table 1**.

Line 173-4, what does it mean ‘systematically selected per district to ensure equal coverage of sites across sub-districts’? ‘30 SAC are sampled per school’: this sample size is pretty low.

- Systematic selection selects at least one school per sub-district to ensure equal coverage across a district. We have clarified this point in the methods (lines 184–85).

As with the traditional sampling, we simply modelled the guidelines of SPPA sampling as referenced in the manuscript (line 188), which included two-phases: district-level sampling of 15 sites/30 SAC per site, followed by sub-district level sampling of 4 sites/20 SAC per site.

Lines 177-8, What the total population size in each of sub-district? Can results from 4 schools (20 SAC per school) represent the prevalence of the sub-district? How many sub-districts are included here in this study? Again, why four schools are purposively selected from each sub-district?

- We thank the reviewer for these clarifying questions. We would like to reference several comments that were answered above that can help answer this question. The assumed district and sub-district sizes were one-million and 100,000, respectively, with an average school size of 400. The SPPA sampling guidelines are referenced in the manuscript.

Line 185, what the model is used exactly here? Is there an algorithm?
Line 190-1, which data are used exactly here. Four references, which were published in different years, re were cited here. Can the prevalence data from these references be merged together?

- We developed a bespoke model for this specific study in R (R version 4.3.2). The districts and the respective sampling strategies were mathematically simulated. The estimated prevalence was dependent on the modelled test sensitivity and specificity (which varied across scenarios) and was compared to the underlying true prevalence in the simulated districts. 1. We have made the model publicly available on Figshare (DOI: <https://doi.org/10.6084/m9.figshare.27325005.v1>.)

The prevalence studies conducted in Kenya and referenced in the manuscript informed our prevalence ranges as shown in **Table 1**—meaning our simulated ranges were realistic and plausible based on the empirical data but were not simulated directly from the data. Our aim was to provide a broad range of possible prevalence distributions.

*Reviewer 2*:

This part is well-organized and more elaborative component of the manuscript. However, study setting and study duration were not described included. The authors should portray specifically where and when the study was conducted. Actually, the specific sites of the study should be elaborated in each sampled schools. The authors have redundantly been using the terms ‘district/sub-district’ throughout the manuscript. What are they referring to with these terms? Perhaps for such ambiguous terms and others, authors may be expected to add operational definition of terms.

It’s not clear why objective of the study was reflected under this part (Ln#121-128). Unless this is the format of the target journal, implicit description of the objective under the last paragraph of the introduction part seems adequate.

- We thank the reviewer for their comments. A district is synonymous with a county (administration level two) and a sub-district is synonymous with a sub-county (administration level three) and we have clarified this point in the methods (lines 199–200). The district size was roughly parameterized to publicly available demographic data from Kenya (lines 198–199). District archetypes, sampling strategies, and diagnostic techniques were all mathematically simulated in R version 4.3.2. No diagnostic sampling took place in any real-world setting, modelling data and outcomes were simulated.
- As multiple reviewers have pointed out the repetition of the study objective in the methods, we have removed this restatement and have clarified the objective at the end of the introduction section (lines 118–124).

*Reviewer 3*:

The methods section is clear and reads very well. Only minor clarifications that could potentially improve the section:

Cost data – You mention Resource use was determined through interviews. Did you use budgeted expenses or executed expenses in your analysis?
Would be good to understand how did you build your district archetypes. Any underlying assumption, previous studies or simply defined by you?
You mention that prevalence studies conducted in Kenya informed the prevalence you defined for districts. Could you be more specific and detail how it informed? Did you simulate district prevalence that are similar to those found in Kenya?

- Thank you for the comment. The costing analysis followed an ingredients-based approach to determine resource use at a district-, school- and child-level. This was informed by consultations with experts in the field currently conducting the field evaluation. Actual prices were applied where available, while budgeted prices were used in cases where actual expenditures were not accessible (and for items that did not significantly drive costs). Additional clarification has been added to the costing paragraph in the Methods.

The model was developed specifically for this study and was built using R 4.3.2. District archetypes were simulated as data frames, which were then called and sampled to model the sampling strategies, and diagnostic techniques (based on test sensitivity/specificity). Prevalence of sub-districts in each district archetype was randomly selected from beta distributions with the upper and lower confidence interval targets outlined in Table 1. School prevalence was then normally distributed around the sub-district mean with standard-deviation 0.0075. We have made the modelling code and data sets publicly available on Figshare: (DOI: <https://doi.org/10.6084/m9.figshare.27325005.v1>.).

The empirical data from the prevalence studies referenced informed the bounds of the beta distributions by suggesting plausible prevalence ranges, but were not meant to be replicates. These studies were more informative for the prevalence ranges in districts with heterogeneous distributions. Overall, our distributions are assumptions informed by the data. Our aim here was to provide a broad range of prevalence levels and distributions to be able to understand how the sampling strategies and testing technologies would perform across different settings.

**Results**

*Reviewer 1:*

Lines 242-49
Which methods were used to obtain these results? How many sub-districts (n=?) How many districts were tested? Are there results based on modelling?

- We apologize for the confusion. All results are based on the model simulations, aside from the cost data that was collected. To make this clearer in the manuscript, we added an additional sub-heading “Modelling Results”, which refers to the “*Test performance*”, “*Comparison of sampling strategies*”, and “*Minimum and optimal diagnostic specifications*” that have now been italicized. The Economic analysis serves as its own section and combines the modelling and costing results to calculate final costs based on modelled outcomes. We have revised the last paragraph of the methods section to clarify the modelling approach (lines 213–222).

As specified in the methods, there are eight district archetypes, with 10 sub-districts each, that were simulated 1000 times. The results presented in the figures are the average outcomes from the 1000 simulations per district archetype. For example, in Figure 3, when we say 80% of sub-districts received the correct treatment decision, it corresponds to an average of 8 sub-districts across the simulations for that archetype because each district had 10 sub-districts.

*Reviewer 2:*

Generally, the report efficiently correlates results with current few literatures, so boosting the study's scientific merit.

The results are well presented and effectively highlighting the comparison of an alternative approach with the conventional diagnostic methods. The issue of cost effectiveness has been the major pillar in disease control including schistosomiasis and the present findings have underscored this scenario. The results were so comprehensive that the outcome might be translated into practice for schistosomiasis elimination policy in sub-districts and specific hot spot transmission sites across the world. On the other hand, overemphasizing of data (numerical data presentation both in text and table/fig.) has been observed in this part. So, authors need to avoid duplicate data presentation. Please check and at least minimize all duplicate presentations both in text and table/figure.

- We understand the reviewers point about the repetition of data points between both the figures and text. We have removed numerical values in the results text that already appeared in the figures and tables (pages 10-12) and adjusted **Figure 1**.

**Discussion/Conclusions**

*Reviewer 1:*

This modelling analysis comes with many important limitations, such as oversimplification. Thus the conclusion drawn from the modelling analysis may be far from accuracy and reliability.

- We thank the reviewer for sharing their interpretation. However, while some aspects of our model, such as the mechanisms to distribute prevalence homogeneously or heterogeneously in the simulated districts can be interpreted as simplistic (as mentioned in the limitations), the goal of this analysis was not to provide exact numerical estimates of SCH surveillance but provide insight on the dynamics and trends between the variables included in the model. In this way, we can provide guidance on the design and implementation of SCH surveillance strategies as new diagnostic technologies become available in many settings. Second, as we highlighted in the limitations section (lines 600–603) we conducted a sensitivity analysis around this particular assumption in which we bimodally distributed prevalence in the districts (which we believe to be a better representation of reality) and saw similar results (**Fig S2**). With respect to the interplay between district prevalence/distribution, test specifications, and sampling strategies, we believe the themes we discuss in this analysis to be credible and our conclusions to be sound. We should also not discredit the other half of our study, which focuses on the costs to implement a CAA RDT through these sampling strategies (costs were collected, not modelled). We argue that a new RDT like that of the CAA would be better suited for more intensive sampling strategies like SPPA (due to decreased costs), which can better estimate prevalence (also observed in [36]) and therefore lead to more efficient drug distribution. As SCH elimination programs look to scale up MDA and need better diagnostics, we believe the CAA RDT could become a valuable tool to these programs.

*Reviewer 2:*

The discussion part was well organized but less emphasis was given on associating similar/related previous reports. The authors were obsessed in interpreting the outcome of their data hich is not discussion. No single citation was there in the first three paragraphs. See paragraph 1, 2 and 3 Ln# 299-336.

-	For instance, the first paragraph is purely method part. So, needless to keep it.

-	The second paragraph is partly a repetition of results with minimum interpretations.

-	The remaining paragraphs are well written.

The conclusion looks interesting. These findings suggest promising innovative diagnostic approach after effective field performance evaluation is practiced. However, I am wondering that why the authors reserve performance evaluation of CAA RDT in the field for recommendation. It would have been an excellent work and complete picture if field performance evaluation of the diagnostic tool was conducted and complemented to this piece of work.

- We thank the reviewer for their comments and interest in the implications of this study. We also look forward to the results of the field performance evaluations of the CAA RDT, and so long as specifications are up to expectations (and TPP requirements), our study can provide contextual evidence for implementation.

Based on the comments of reviewer 2 and reviewer 3 we have revised several paragraphs in the discussion section and, as suggested, removed the repetition of some results. We also sought to contextualize our results with additional studies from the literature, as suggested. However, the vast majority of SCH modelling focuses on SCH transmission, which is of a different nature than what we present here.

*Reviewer 3:*

The first three paragraph broadly repeat the results section. While always a good practice to revisit main results, it looks a bit excessive, particularly considering the detail and objectiveness of the results section. I would advise reducing condensing these two paragraphs to focus the discussion more on the broader comparison with other studies, the usefulness of results and limitations of the study.

Line 377 – it also depends on quality and capacity of diagnosis of microscopists. Would be good to expand your discussion on this by adding references but also highlighting how this test somehow removes the effect of the quality/experience of the microscopist

It is not clear if this new test provides semi quantitative results in any shape or form. When you mention the value for impact assessments, it should be clear that reduction elimination of SCH as a public health problem still depends on quantitative measures as the indicator is measured through the prevalence of heavy infections, which are based on egg counts, and not overall prevalence. My suggestion here is to clarify that this tool can still be a plus for measuring prevalence and target treatments but has its limitation to assess elimination achievements. I understand the WHO guidance for attributing EPHP may be an issue but it is the criteria we have. If the new test provides some sort of quantitative approach. This idea is stated in Background and then again in Conclusions where you state progress towards elimination but then mention SCH prevalence mapping. It should be clear that these tests, as they are, have their own limitations to assess SCH elimination achievements

- We thank the reviewer for their comments, interpretation and suggestions. Based on your comments and those from reviewer 2, we have revised several paragraphs throughout the discussion section to incorporate these suggestions, notably contextualizing our results with additional studies from the literature. With respect to quality and capacity of microscopists, while we do agree there is space for human error in Kato-Katz/urine filtration, it seems the larger pitfalls of microscopy lie in sample consistency and infection intensity. We have added commentary on this with respect to the potential role of a CAA RDT (lines 361–369). On a similar topic, we also addressed the need for semi-quantitative tests to assess elimination goals.

To clarify our thought process with respect to elimination: elimination programs and guidelines aim to increase MDA to reduce prevalence and infection intensity down to elimination targets. This will require a larger supply of PZQ or more efficient use of PZQ. Precision mapping strategies like SPPA can lead to more efficient use of PZQ through more accurate estimation of prevalence. The CAA RDT can then better support SPPA sampling through reduced costs/labor, with the additional benefit of higher sensitivity in low prevalence settings (presumed). We hope to have better articulated this perspective in our revised discussion.

**Summary and General Comments**

*Reviewer 1:*

The study by Chevalier aims to evaluate the impact and cost of a new rapid diagnostic test, POC-CAA, for school-based prevalence mapping and monitoring and evaluation surveys of schistosomiasis using a modelling analysis. While the topic is of interesting, the manuscript suffers from some weaknesses. The methods section is still very elusive, with lots of key information missing. The rational for the modelling analysis is unclear. In addition, the modelling has lots of limitations as stated in the discussion section such as oversimplification, indicating that the conclusions drawn from a simplified modelling may be far from accuracy and reliability. In the title, it states that this is a modelling study. However, no mathematical model has been mentioned in the Abstract and Introduction sections.

- Throughout this revision and through our responses to the other comments provided, we hope to have sufficiently addressed these concerns and clarified our modelling methodology and rationale. As the last sentence of our introduction now states: “This analysis seeks to provide evidence on the interplay between costs, test performance, and sampling accuracy to guide test adoption decisions, especially given recommendations for new sampling strategies.” Our model provides insight on how test sensitivity and test specificity will affect the estimated prevalence through these alternative treatment strategies and therefore impact treatment outcomes. This evidence can be used to understand what impact the CAA RDT will have on prevalence surveys once we know what the sensitivity and specificity of the test will be.

Abstract section
1. Background: The last sentence sounds a conclusion. A prototype circulating anodic antigen rapid diagnostic test (CAA RDT) could replace the standard tools for mapping (Kato-Katz and urine-filtration) and better support precision-mapping and thus more efficient drug distribution.

2. Methodology: The section is descriptive, without any detailed methods provided, e.g., sample size? The first sentence is very long and difficult to follow.

3. Results: some sentences are like conclusions: In settings with both S. mansoni and S. haematobium, a CAA RDT with comparable performance to Kato-Katz/urine filtration would be cost-saving. Further,
the use of a CAA RDT for M&E would be the most cost-effective strategy as compared to two consecutive day sampling required with Kato-Katz.

- We thank the reviewer for their input. The abstract section has been revised.

Author Summary
Line 69, Using a mathematical model. Which model? Why the model used here can achieve the purpose of the study? What’s the rational of the modelling?

- We do not believe this to be relevant to the author summary. Instructions for the author summary are as follows: “a 150–200-word non-technical summary of the work...should highlight where the work fits in a broader context of life science knowledge and why these findings are important to an audience that includes both scientists and non-scientists.” As our model is a bespoke model (i.e., not an easily referenced existing modeling framework), we do not think it is reasonable to describe the model and rationale behind its design in a brief, non-technical summary.

We hope to have sufficiently addressed the model rationale through our other responses and revisions in this letter.

Introduction section
Line 90, Can the author provide more details about: ‘often characterized by heterogeneous geographical distribution’? What’s the geographical level? District? Sub-district? Village?

- As schistosomiasis is focal and depends on proximity to waterbodies, as well as the contact patterns of individuals that live near those water bodies, the heterogeneous distribution of prevalence could range from village to village, sub-district to sub-district, or district to district. In the introduction we were highlighting this feature of schistosomiasis. We have clarified this point in the introduction (lines 91–92) and added an additional reference for context.

*Reviewer 2:*

**Title:** It is acceptable in its existing form but better if redundancy of using ‘evaluation’ is omitted.

Perhaps the authors should consider the following minor and/or line by line comments:

- Misplacement of items like objective under methods part is not common practice unless the rule of target journal. Otherwise, objectives are indicated implicitly under the last paragraph of the introduction.
- Scientific names should strictly be italicized and with consistent spacing throughout the manuscript. see abstract (results) and pp.14 Ln # 311
- Revise some abnormally long sentences Ln # 46-49, 97-100, 331-334, 370-373.
- Mixed referencing style (author-year Vs numeric citation style). Address those inconsistencies of citations in the text. See pp. 15, 16, 17 Ln # 356-357,361,386-387. Should be as follows: Knowles et al. (23)…, Finally, Worrell et al. (3) as cited in Ln#151…
- References are relatively too few. Hence, pertinent and up-to-date references should be added to enhance the visibility of the study.
- Improve the quality and clarity of figure 3 with regard to labeling. Table and Figure legends should be self-standing. Table legends do not need full stop see Table 1.
- Please check and follow the reference pattern as per journal and also balance of references in text Vs reference site.

Overall, the manuscript is well-structured, with a clear methodology and language command as well as compelling results, making a valuable contribution in mapping of schistosomiasis for possible elimination of the disease. I recommend acceptance with minor revision.

- We thank the reviewer for their keen eye and editorial suggestions. We have attempted to address all comments listed above. And finally, we thank the reviewer for their positive interpretation of our study.

*Reviewer 3:*

This is an interesting paper that measures several key components essential to measure progress of SCH programs towards elimination. New diagnostic tools are key to keep progress as well as assessing the best sampling strategies and comparing the most cost effective mapping approaches. Well done in doing it as it provides a very interesting modelling exercise with clear data and values that can be useful for decision making, particularly to decide on the costs of traditional vs oversampling approaches.

The main weakness of the paper, in my opinion, is it lack of translation into practical public health measures. The discussions mostly repeats the results and does not provide a broader discussion about the implications of these results: what should program managers do with these results, what further research should be done to validate these results with real field data? Any recommendations for WHO to adjust sampling procedures and/or diagnostic approaches? The paper misses the research translation element that is key to put research into practice and would certainly improve if such element is added.

- We understand the perspective shared by the reviewer here. However, we think the translational aspect is dependent on several other pending factors that are outside of our control. For example, the SCH field, including the WHO, is already advocating for a shift to precision mapping strategies (SPPA). What our study does provide is additional evidence that SPPA sampling will be a more effective sampling procedure and implementation can be made more affordable through the use of an RDT like the CAA. This will be an important incentive in promoting the strategy in relevant countries. However, this all hinges on the CAA RDT having good and reliable performance in the field, which we do not yet know (field trials are actively underway)—although in-lab evaluations show promise. The additional aspect of this is whether the CAA RDT can serve as a semi-quantitative test and indicate infection intensity through CAA concentration, which we now address in the discussion. Overall, we hope this messaging comes across more clearly in our revised manuscript. Finally, we thank the reviewer for reviewing our study and providing insightful and constructive commentary.

We again thank the reviewers and editorial team for their time and commitment to reviewing our study. We hope to have sufficiently addressed all feedback and hope that our work can contribute to the schistosomiasis literature through publication in *PLOS Neglected Tropical Diseases*.

On behalf of all authors,

Joshua M Chevalier

j.m.chevalier@amsterdamumc.nl

Sarah Girdwood

Sarah.Girdwood@finddx.org

Kyra Grantz

kyragrantz@gmail.com

Shaukat Khan

sakhan@gmail.com
