# Supplementary material for: The impact and cost of a new rapid diagnostic test for school-based prevalence mapping and monitoring and evaluation surveys of schistosomiasis: A modelling study
Source: PLoS Negl Trop Dis. 2025 May 12;19(5):e0013071. doi: 10.1371/journal.pntd.0013071 (PMC12097705; doi:10.1371/journal.pntd.0013071)
Supplement: Attachment — Submitted filename: PLOS_NTDS_R&R2_2025Apr08.docx [file pntd.0013071.s005.docx]

**15 April 2025**

Dear editors and reviewers,

We would like to thank you again for the opportunity to revise our study for publication in *PLOS Neglected Tropical Diseases*. Please find below our detailed responses to all reviewer comments.

**Methods**

Reviewer #1: No subtitle for the first paragraph. Still it should not belong to this section.
Ln 131, Ethics statement
Ln 135, Diagnostic technologies
Ln 155, what does KEMRI stand for?
Ln 169, Sampling strategies

- **Response**: We thank you for sharing your perspective on the structure of the methods. We have moved the first paragraph from the beginning of the methods section to a new sub-section at the end of the methods titled “Outcomes” (line 217). Additionally, we have specified the acronym KEMRI stands for Kenya Medical Research Institute in lines 146–147.

Ln 270-3, We simulated eight different district archetypes, each consisting of 10 sub-districts with 50 schools per sub-district, with varying prevalence distributions. District size, with respect to number of sub-districts and primary schools per sub-district, was parameterized to demographic data from Kenya [15–17].
However, the rational for this simulation is unclear in the main text but in the response to my previous comment #3, i.e., the district size in this analysis would correspond to a moderately sized district of one-million people, divided into 10 sub-districts of roughly 100,000 people each (based on 2019 census data). We assume 20% of the population (20,000/sub-district) is school-aged children, divided into schools of an average size of 370 (based on the Kenya Demographic and Health Survey of 2022 and the Basic Education Statistical Booklet of 2020), rounded to 50 schools per sub-district. These details should be included in the main text to increase the logicality, readability, and reproducibility.

- **Response**: We agree this would provide clarification and have added the suggested editions in lines 189-192.

In addition, to ensure transparency and reproducibility of the study, the authors should mention that the model has been publicly available on Figshare (DOI: <https://doi.org/10.6084/m9.figshare.27325005.v1>.) in the main text or attach the model as supplementary data, not just in the response to reviewers’ concerns.

- **Response**: We agree that the data and model code should be publicly available with the publication for reproducibility. We have provided *PLOS Neglected Tropical Diseases* with a data availability statement (and the Figshare link) in the submission system. This will be published along with the manuscript. This statement does not currently appear as part of the manuscript’s main text.

Table 1 should be cited after the sentence: District archetypes included a low prevalence district (average prevalence 5%); moderate prevalence districts near the 10% prevalence threshold (8% and 12%), and a high prevalence district (20%), all with either a homogeneous or heterogeneous distribution of sub-district prevalence.

- **Response**: Table 1 has now been cited in line 196.

Ln 206-7: Prevalence among children at each school within a sub-district is assumed to be homogeneously distributed (standard deviation of 0.75% around the mean sub-district prevalence).
How about the prevalence among children at each school within a sub-district is assumed to be heterogeneously distributed?

- **Response**: Thank you for your feedback and we agree this is an important question. With respect to the focal nature of schistosomiasis, this was an assumption we made to represent the idea that schools within a region are likely to have similar prevalence levels due to the geographic features of the region (such as proximity to water bodies). We have run additional analyses to understand this effect and we now discuss this in greater detail in the discussion section:

“Similarly, all district archetypes assumed homogenously distributed prevalence among schools within a single sub-district due to proximity to geographic features (such as water bodies). In a district consisting of 10 sub-districts, traditional sampling would select 0.5 schools per sub-district on average while SPPA sampling would select 1.5 schools, so if school prevalence varies widely within a single sub-district, neither strategy is well equipped to accurately estimate sub-district prevalence. In such regions, when heterogeneity is suspected, SPPA guidelines recommend progressing directly to precision assessment, which samples 4 schools per sub-district and may be more adept at estimating sub-district prevalence.” (lines 396–402)

**Results**

Reviewer #1: Ln 228, Difficult to follow the sentence:
The cost of testing a district using traditional sampling with the CAA RDT was on par with the cost of testing using ?(Fig 1)

- **Response**: Thank you for pointing this out, the line has now been revised. It reads: “The cost of testing a district using traditional sampling with the CAA RDT was slightly less than the cost of testing using Kato-Katz (**Fig 1**).”

Fig 1. It is difficult to observe the number in dark purple bar.

- **Response**: Thank you for pointing this out, we have corrected the coloring in **Fig 1**.

Ln 242-3, for phase 1 was only 8–12% more expensive per SAC than traditional sampling across both diagnostics, but if phase 2 was warranted, the cost per SAC was considerably higher than under the traditional sampling method (113–139%).
How to get these percentages? Could you check again? Also check the percentages (3-12% less) in Ln 245, and (5-7% less) in Ln 246.

- **Response**: We thank you for raising this point and have double checked these calculations (the percent difference between cost/SAC as shown in **Fig 2**). Indeed, the percentages from lines 242-243 were outdated, they should read “for phase 1 was only *8–20%* more expensive per SAC than traditional sampling across diagnostics, but if phase 2 was warranted, the cost per SAC was considerably higher than under the traditional sampling method (*64–97%*)”. We sincerely apologize for this oversight and thank you, again, for this correction.

From line 245, **Fig S1** in the supplement shows the percentage cost difference between SPPA and traditional sampling when staffing and/or transport costs are halved, as is being referenced here.

Ln 253, Modelling results
Ln 254, Test performance

Fig 4 the symbol legend is a bit confusing

- **Response**: We have modified the figure caption for clarification (line 280).

Ln 303, $3,891 if traditional sampling is used relative to Kato-Katz of $4,240.

**Conclusions**

Reviewer #1: The limitations of analysis are clearly described.

Ln 620, We have shown that a CAA RDT with a high specificity will be
Maybe mention the model here to void confusion.

- **Response**: We have followed your suggestion, and the line now reads “This modelling analysis indicates that a CAA RDT with a high specificity will be a valuable cost-competitive diagnostic tool to be used in SCH prevalence mapping, particularly under precision mapping strategies.” (line 429)

**Summary and General Comments**

Title: The impact and cost of a new rapid diagnostic test for school-based prevalence mapping and monitoring and evaluation surveys of schistosomiasis: a modelling study

Ln 38: In endemic communities where the prevalence of schistosomiasis

Introduction:
References are missing for a number of sentences in the Introduction section, such as:
A circulating cathodic antigen (CCA) rapid diagnostic test (RDT) has been commercially available as a point-of-care test since 2008, however, despite being easier to use and less labor intensive than Kato-Katz, it only works well for detecting S. mansoni infections and its sensitivity decreases with decreasing infection intensity.

A new RDT, (still under-development), can detect circulating anodic antigen (CAA) in an infected host’s blood, as a marker for active infection for S. mansoni, S. japonicum and S. haematobium.

Based on early evaluations, the CAA RDT prototype has the potential to reliably measure prevalence in SAC as well as in adults.
Ln 311, As schistosomiasis
Ln 315, a novel rapid diagnostic test in the CAA RDT?
Ln 323, the cost of testing a district using Kato-Katz ($3,035 v. $3,307,
Ln 324, the CAA RDT at $2,678 per
Ln 327, all three Schistosoma (italic)

- **Response**: Thank you for pointing these out, we have updated references where indicated. (lines 106-109)

Ln 331-2, Which result supports this statement: In a sensitivity analysis, staffing and transport costs were halved to approximate costs that would be applicable to smaller regions such as the Philippines where S. japonicum is present, noting that lots of parameters used in the model were based on the Kenyan data. In addition, the transport costs would not be cheap in the Philippines as goods were transported by airplane between islands. Why the regions in the Philippines are smaller?

- **Response:** Thank you for your feedback. Staffing and transport costs were halved in the sensitivity analysis as presented in **Fig S1** in the supplementary appendix. As the Philippines represents a smaller geographic region than Kenya, the distance between sampling sites within a single district is assumed to be smaller by comparison. Additionally, we assumed the cost for testing a single district, which would not require plane transport between islands. We present the Philippines as an example of a smaller country in which transport costs may be less in our sensitivity analysis, but actual costs were only estimated from Kenya-based sampling.

Ln 369, Additionally, in [35]
Is it the journal’s style to cite a reference. The same issue applies to Ln 378 and Ln 383.

- **Response**: The references have been corrected in lines 371, 381, 386.

The first three paragraphs in the discussion section remain repetitive to background and result sections.

- **Response**: We appreciate your feedback on the discussion section. We have revised and reduced the first three paragraphs of the discussion section (line 313) to avoid repetition.

We want to express our appreciation to the editors and reviewers for taking the time to review our study and provide important feedback that strengthens the manuscript. We hope to have sufficiently addressed all reviewer comments and look forward to hearing your response.

Sincerely,

On behalf of all authors,

**Joshua Chevalier	Shaukat Khan**

j.m.chevalier@amsterdamumc.nl	sakhan@gmail.com
